# Editorial: Psychiatric comorbidities in patients with epilepsy: diagnosis and treatment

**DOI:** 10.3389/fpsyt.2023.1217656

**Published:** 2023-06-06

**Authors:** Silvia Oddo, Mercedes Sarudiansky, Lorna Myers, Luciana D'Alessio

**Affiliations:** ^1^Epilepsy Center, El Cruce Hospital and Ramos Mejía Hospital, Studies in Neurosciences and Complex Systems-National Scientific and Technical Research Council (ENYS-CONICET), Buenos Aires University, Buenos Aires, Argentina; ^2^Faculty of Psychology, National Scientific and Technical Research Council (CONICET), Buenos Aires University, Buenos Aires, Argentina; ^3^Department of Clinical Psychology, Psychogenic non-epileptic Seizures Program, Northeast Regional Epilepsy Group, New York, NY, United States; ^4^Epilepsy Center, Ramos Mejía Hospital, Institute of Cell Biology and Neurosciences-National Scientific and Technical Research Council (IBCN-CONICET), Buenos Aires University, Buenos Aires, Argentina

**Keywords:** psychogenic non-epileptic seizures (PNES), functional dissociative seizures, functional neurological disorders, depression comorbidity in epilepsy, psychosis comorbidity in epilepsy

The scientific field is increasingly recognizing the importance of psychological and psychiatric health in persons with epilepsy. Recent definitions of epilepsy from the ILAE (International League Against Epilepsy), include behavioral comorbidities and conceptualize epilepsy as a disorder of the brain, characterized not only by epileptic seizures but also cognitive and neurobiological factors (1). Furthermore, in 2017 the ILAE suggested that in the earliest diagnostic stages, emotional disorders should be considered, allowing treatment of epilepsy and comorbidities to be initiated in the most timely manner (2).

Studies have demonstrated in recent years that psychiatric comorbidities are commonly observed in patients with epilepsy, especially among those with pharmacoresistant epilepsies such as treatment resistant focal temporal epilepsy and focal extratemporal epilepsy (e.g., frontal lobe) (3, 4). It is estimated that psychiatric symptoms and disorders including depression, anxiety, psychosis, impulsivity, and personality, somatoform, and functional neurological disorders [which include psychogenic non-epileptic seizures (PNES), also known as functional dissociative seizures (FDS) or functional seizures (FS)] can affect 20–50% of patients with treatment resistant epilepsy (5–10). Additionally, around 20–30% of those seen in an epilepsy center are diagnosed with PNES/FDS (8, 11, 12).

In these cases, the presence of psychiatric comorbidities, can condition therapeutic decisions and antiseizure treatment choices. Moreover, behavioral symptoms affect the patients' quality of life and in some cases, can even have a greater impact than that of the actual epileptic seizures (4, 13). In addition, it has been suggested that the presence of psychiatric comorbidities can affect the prognosis of epilepsy, especially in those who have undergone epilepsy surgery (14, 15). Sadly, psychiatric comorbidities tend to be underdiagnosed and undertreated; timely detection would produce better prognoses. It is for these reasons that multidisciplinary studies of these comorbidities are recommended (15).

In this special edition of Frontiers in Psychiatry, we have been given the opportunity to present novel and interesting reports on the following topics:

Malik et al.'s study showed how stigma and psychosocial factors, affect epilepsy patients and are associated with high levels of depression, anxiety and poor quality of life. This report underscores the importance of multidisciplinary health systems that include psychologists, psychiatrists, primary care and specialized physicians, who can work together to achieve de-stigmatization and increased knowledge about epilepsy through community-based educational initiatives.

Paredes-Echeverri et al. highlighted the significant role that psychological trauma and early adverse experiences have on the pathophysiology of certain severe FND subtypes. Based on the emerging neurobiological discoveries and the original data the authors discuss, they propose an FND subtype that significantly impacts our understanding of the general treatment of PNES/FDS and may help tailor psychotherapeutic approaches.

Krámská et al. compared attachment styles of primary caregivers (types and quality) in patients with PNES compared to healthy volunteers in the Czech Republic. They found that patients with PNES reported greater levels of maternal/paternal overprotection and that the most common attachment style was anxious-ambivalent (type 2). The authors posited that this could be useful for differential diagnoses and to refine treatments for PNES.

Similarly, Roberts et al. investigated the traumatic comorbidities and socio-emotional processes in patients with PNES/FDS. The authors examined a community sample of participants diagnosed with PNES/FDS and who had developed post-traumatic stress (PTS) and found that those with clinical levels of PTS (FDS-PTShi) presented with greater emotional avoidance and dysregulation and more perceived stress than those with low clinical levels of PTS (FDS-PTSlo). Furthermore, participants with FDS-PTShi reported less reappraisal, more loneliness and less emotional contact than the other group. These findings suggest that fomenting significant connections with others, including through emotional contact, can be an important objective in treating those with PNES/FDS and PTS.

Doss focused on PNES/FDS in a pediatric population. She explored the psychiatric antecedents in families and found that parents seemed to be uninformed about their children's psychiatric/psychological symptoms and a significant number of first-generation family members had experienced psychiatric disorders themselves. This supports the importance of approaching the patient's treatment from this familiar orientation.

With regard to psychosis in epilepsy, a very important topic due to how grave this presentation can be in its acute phase, and which is understudied and poorly understood, Sone contributed a mini review narrative of the most recent findings in neuroimaging and discussed their importance. Neuroimaging represents a useful tool for investigating the human brain and psychiatric disorders. Certain findings such as reduction of white matter and hyperactivity in temporo-frontal areas observed in functional studies are associated with psychosis in epilepsy which suggests the existence of affected neurobiological mechanisms that remain unexplored and misunderstood.

As for treatments, a novel and interesting mini-review by Epps has granted us the opportunity to synthetically and yet comprehensively consider the possible pathophysiological mechanisms of depression in epilepsy, and to consider the plausibility of using cannabis derivatives based on known actions of endocannabinoid system. This author examined current literature and considered the possibility that these agents could serve a dual purpose in the treatment of epilepsy and of associated depressive symptoms.

The aim of the Research Topic was to bring together current quality articles from researchers working in the area of epilepsy and mental health focused on psychiatric comorbidities in epilepsy and psychogenic non-epileptic seizures or functional dissociative seizures (PNES/FDS). We hope this topic will interest you especially due to its valuable translational significance.

## Author contributions

All authors wrote the editorial together and approved the final version.
